# Psychometric properties and reliability of the Brazilian version of *Parent Attitudes about Childhood Vaccine* (PACV-BR)

**DOI:** 10.1590/1984-0462/2024/42/2023019

**Published:** 2023-09-15

**Authors:** Claudio José dos Santos, Maria Rosa da Silva, Paulo José Medeiros de Souza Costa

**Affiliations:** a Universidade de São Paulo, São Paulo, SP, Brazil.; b Universidade Estadual de Ciências da Saúde de Alagoas, Maceió, AL, Brazil.

**Keywords:** Brazil, Childhood vaccine, Vaccine hesitancy, Validation studies, Brasil, Vacina infantil, Hesitação vacinal, Estudos de validação

## Abstract

**Objective:**

To evaluate the psychometric properties and reliability of the Brazilian version of the tool *Parent Attitudes about Childhood Vaccine* (PACV-BR).

**Methods:**

The sample included 110 parents of children up to two years old served by Family Health Basic Units. The tool's internal consistency and factor validity were respectively assessed by Cronbach's alpha and exploratory factor analysis (EFA). The test-retest reliability was assessed by the intraclass correlation coefficient (ICC).

**Results:**

The EFA results indicated a proper structural adequacy of the PACV-BR (15 items and two factors). The reliability generated Cronbach's alpha values between 0.715 and 0.854 for the items, of 0.918 for the tool as a whole, of 0.877 for factor 1 and of 0.825 for factor 2, in addition to an ICC of 0.984.

**Conclusions:**

The PACV-BR showed evidence of construct validity and reliability.

## RESUMO

**Objetivo:**

Avaliar as propriedades psicométricas e a confiabilidade da versão brasileira do instrumento *Parent Attitudes about Childhood Vaccine* (PACV-BR).

**Métodos:**

A amostra incluiu 110 pais de crianças de até dois anos atendidas em Unidades Básicas de Saúde da Família. A consistência interna e a validade fatorial do instrumento foram avaliadas, respectivamente, pelo alfa de Cronbach e pela análise fatorial exploratória (EFA). A confiabilidade teste-reteste foi avaliada pelo coeficiente de correlação intraclasse (ICC).

**Resultados:**

Os resultados da AFE indicaram adequação estrutural do PACV-BR (15 itens e dois fatores). A confiabilidade indicou valores de alfa de Cronbach entre 0,715 e 0,854 para os itens, de 0,918 para o instrumento como um todo, de 0,877 para o fator 1 e de 0,825 para o fator 2, além de ICC de 0,984.

**Conclusões:**

O PACV-BR apresentou evidências de validade de construto e confiabilidade.

## INTRODUCTION

Vaccine hesitancy — defined as the delay to accept or refuse vaccinations, despite its availability in the immunization services^1,2^ — is a phenomenon that gained such proportions it was classified by the World Health Organization as one of the greatest ten threats to world health with which the world nations must deal.^3^

One of these initiatives was the *Parent Attitudes about Childhood Vaccine* (PACV), a tool originally developed by a group of researchers from Washington, USA. It is meant to identify parents that are hesitant towards childhood vaccines and, therefore, to support vaccine education activities. The PACV was originally developed by adapting items of previous studies about health beliefs, submitted to focus groups composed of American parents and a panel of vaccine experts. The result thereof was the development and validation of a tool widely accepted in the USA and throughout the world.^4–6^

Due to its relevance, since its development, several papers validated and used the PACV in Europe and in other countries. In Brazil, the PACV went through the processes of transcultural adaptation to Brazilian Portuguese and face and content validity. This version was adapted to the national context and called PACV-BR.^7^ However, the PACV's Brazilian version still was not submitted for structural validity and reliability analysis.

In this study, our objective was to assess the psychometric properties and reliability of the PACV tool's Brazilian version.

## METHOD

This is a methodological study of construct validity and reliability analysis of a tool previously adapted to the Brazilian cultural context.

The PACV-BR has 17 items, including two sociodemographic characterization items and 15 others that are added in a Likert scale to obtain the tool's final score — which ranges from 0 to 100. The tool has two types of closed-ended questions: Likert-type scaling, of five points, and dichotomous questions with two/three answer possibilities. In average, it takes 5 minutes to fill the PACV-BR, and its text complexity is at the secondary school level. The PACV-BR score is obtained as follows: two points are attributed to “hesitant” answers, one is attributed to answers that reflect “uncertainty,” and zero points are attributed to “non-hesitant” answers. The gross score is calculated by the simple sum of the items and then those points are converted into a score ranging from 0 to 100 using a table made by simple linear transformation.

This is a study carried out with individuals of both genders, the parents of children from zero months to two years of age, registered with the Family Health Strategy. This age group was selected because it represents an appropriate period to get parents’ perspective on the vaccines that are part of the Brazilian Immunization Schedule, which are usually applied in the first 15 months of the child's life, with boosters given up to their fifth year. Additionally, this period was chosen because it is when the children get the doses that usually make the parents question, delay, or refuse them.

The individuals registered with the Family Health Strategy were chosen because family health is a reorientation strategy of the assistance health model in Brazil, operated by multiprofessional teams responsible for monitoring a set number of families located in a specific geographic area. Additionally, the Basic Family Health Units (UBSF, an acronym in Brazilian Portuguese) are the reference spaces of the Unified Health System (SUS, in Brazilian Portuguese) in the national territory to immunize the population for free in vaccination rooms located throughout the country.

To determine the sample size for validity studies, the proportion of 1:5 to 1:10 of each item to be validated by the participants is recommended.^8^ Therefore, a sample size of 85 parents was necessary. Considering the possibility of quitters and incomplete forms, 85+(20%)=102 was the minimal size of the sample. Therefore, 110 collections were planned.

To select the sample, the following methodology was adopted: all the sanitary districts of a capital in the Northeast of Brazil were numbered for random selection. Once the health district was selected as part of this research, the UBSFs were identified in the territory's encompassing area. Three (50%) of the total of six UBSFs of the district were randomly selected to be a part of this research. In each UBSF selected, the total of children between zero months and two years of age was counted in the UBSF's encompassing area through e-SUS Primary Care. The proportion of children between zero months and two years by unit was calculated in comparison to the total of children between zero months and two years who were part of the universe of the three UBSFs selected for this study. With this proportion, the number of parents of children in that age span, by UBSF, who were supposed to be part of the study was obtained. Therefore, from the three UBSFs selected, the following proportions of individuals were set: 32% (35), 43% (47), and 25% (28), totalizing 110 parents of children between zero months and two years established in the sample planning, according to the sampling process of the study shown in Table 1.

**Table 1:** Study sampling process.

	Basic unit I	Basic unit II	Basic unit III	Total
Registered children (N)	532	725	410	1,667
Registered children (%)	32	43	25	100
Sample per unit	38	52	30	120

To select the parents, an alphabetic list was made with the names of the children in the age group of interest that were registered with each UBSF, and the sample was selected by simple randomization using a non-commercial electronic tool specialized in the generation of random sequences. For every selected child, the respective address registered with the reference UBSF was identified and his/her father or mother was personally invited to take part in the research. If at least one of the parents could not be found at home, refused to take part in the research, or if, by any reason, the father and/or mother could not take part in the study, another child would be selected.

The characterization variables of each participant were evaluated by a sociodemographic qualification questionnaire. The parents’ vaccine hesitancy was measured by the administration of the self-applicable PACV-BR tool previously translated and culturally adapted into Brazilian Portuguese. Parents with a PACV score ≥ 50 were classified as having a high probability of vaccine hesitancy. To obtain the status of self-reported dose loss and delayed vaccination, the following questions were asked: “Did you ever fail to take your child to get one of the regular doses of the childhood immunization schedule?” and “Did you ever delay any of the regular doses of your child's immunization schedule?” The parents that answered this question affirmatively were respectively classified as self-reported refusers or hesitant individuals.

For the test-retest reliability evaluation, the tool was once again applied to a random sample of 22 (20%) participants that were part of the tool's first application. This second application occurred one week after the first one.

The software Statistical Package for the Social Sciences (SPSS) version 22.0 was employed. Descriptive statistics were used to illustrate the participants’ demographic data. The sample's adequacy was evaluated utilizing the Kaiser-Meyer-Olkin (KMO) measure and the data's adequacy was assessed using Bartlett's test of sphericity.

We applied exploratory factor analysis (EFA) to decrease and summarize the questionnaire's items in order to form factors and group the PACV's questions. The chosen extraction method was the analysis of main components through the Varimax orthogonal rotation to discriminate the best pertinence of the variables to the identified components. The formation of the factors followed two criteria: the level of association between the variables, obtained through the factor loads (>0.400), and their level of semantic relationship and proximity with the other items of the component.^9,10^

In addition to factor analysis, validity evidence were also investigated based on the technique of known groups through the application the *t*-test for independent samples to compare the PACV-BR's mean scores among the parents with or without the status of self-reported dose loss and/or delayed vaccination. The objective of this analysis was to identify if the PACV's version translated into Brazilian Portuguese was sensitive enough to detect the differences between the two known groups of parents. The significance level adopted was of p-value >0.05.

Reliability was measured through internal consistency analysis and Cronbach's alpha. To represent high internal consistencies, values ≥0.70 were considered trustworthy. This coefficient ranges from 0 to 1, and a value lower than 0.5 is deemed low; values greater than 0.6 are acceptable; greater than 0.7 are good; and greater than 0.8 are great.^10^ The test-retest reliability was evaluated though the calculation of the intraclass correlation coefficient (ICC), interpreted as follows:<0.40 was deemed poor, between 0.40≤ICC<0.75 good; and ≥0.75, excellent.^11^

The study was approved by the Research Ethics Committee of the National Commission for Research Ethics — CEP/CONEP system through Certificate of Presentation for Ethical Consideration — CAAE number 30815420.3.0000.5011.

## RESULTS

One hundred and ten parents participated in the PACV-BR's psychometric property analysis. Generally, the mean age was of 26.8±5.9 years, 50.0% of the sample was married, 56.4% was brown, 87.3% had only one child, 65.5% were mothers, 87.3% had a child between zero and one year old, 67.3% had only one child at home, 49.1% graduated from high school, 82.7% had a religion, 68.2% had a gross family income of up to two minimum wages (R$ 2,090.00) and 56.4% claimed to live in a home with up to three people.

Bartlett's test of sphericity and the KMO adequacy index proved the sample's adequacy (χ^2^=1,491.336; KMO=0.864; p=0.000).

The exploratory factor analysis identified two independent factors for the items that compose the tool. These factors, when added, explain 61.3% of the total variation of the PACV's version adapted to the Brazilian cultural context. Factor 1 (F1) was composed of eight items related to the parents’ attitude to immunization, encompassing behaviors, demeanors, and actions taken by them towards childhood vaccines. Factor 2 (F2) was composed of seven items related to the parents’ beliefs, concerns, and trust towards the vaccines. All items presented a fraction of variation explained by common factors >0.50. Table 2 presents the results of the structural matrix with the Varimax orthogonal rotation of the PACV-BR's factors.

**Table 2:** *Parent Attitudes about Childhood Vaccine* — Brazilian version's factor structure.

Tool items	F1	F2
Item 3	0.553	
Item 4	0.973	
Item 5		0.962
Item 6		0.896
Item 7		0.825
Item 8		0.822
Item 9		0.674
Item 10	0.584	
Item 11	0.827	
Item 12	0.793	
Item 13	0.610	
Item 14	0.869	
Item 15		0.882
Item 16	0.828	
Item 17		0.911
Eigenvalue	6.614	2.591
% explained variation	44.092	17.274
% explained variation total	61.367	
No. of items	15	

F1: Factor 1; F2: Factor 2.

**Figure 1:**
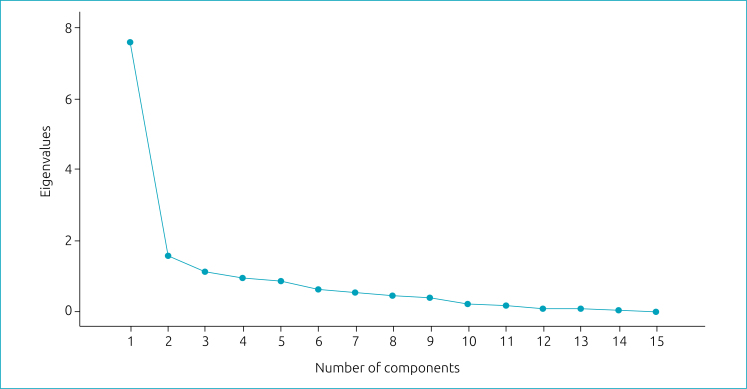
Screen test chart of the eigenvalues obtained by the *Parent Attitudes about Childhood Vaccine* — Brazilian version exploratory factor analysis.

The eigenvalue chart shows the components’ dispersion in the screen test and confirms that the PACV-BR has two factors (Figure 1).

The tool's internal consistency analysis values were: factor 1's total alpha=0.877; factor 2's total alpha=0.825; PACV-BR's total alpha=0.918. The detailed study of the PACV-BR's internal consistency, by item and dimension, can be found in Table 3.

**Table 3:** *Parent Attitudes about Childhood Vaccine* — Brazilian version's internal consistency and test-retest stability.

Factor/dimension	Item	Cronbach's α*	95% CI
	Item 3	0.847	
	Item 4	0.854	
	Item 10	0.850	
Behaviors	Item 11	0.787	
	Item 12	0.803	
	Item 13	0.834	
	Item 14	0.792	
	Item 16	0.801	
	Item 5	0.824	
	Item 6	0.715	
Beliefs	Item 7	0.823	
	Item 8	0.830	
	Item 9	0.802	
	Item 15	0.730	
	Item 17	0.745	
α_total_ Factor 1		0.877	
α_total_ Factor 2		0.825	
α_total_ Tool		0.918	
Intraclass correlation coefficient		0.984	0.963–0.993

* Cronbach's α if the item was excluded.

Based on the composition of the PACV-BR's final scores, the test-retest reliability of the tool's version adapted to Brazilian Portuguese was calculated, obtaining the ICC value of 0.984 (confidence interval — CI95% 0.963–0.993) in the analysis of both applications.

The independent *t*-test showed that, in average, the parents that reported delaying at least one of the regular doses of the childhood immunization schedule had PACV-BR scores greater than those of the parents that did not report said behavior (*p*=0.000). Likewise, the parents that reported missing one of the doses of the regular schedule also presented PACV-BR scores greater than those of the parents that did not report this behavior (*p*=0.000). This analysis is shown in Table 4.

**Table 4:** Student's *t*-test for the comparison of *Parent Attitudes about Childhood Vaccine* — Brazilian version score means obtained by parents a) with and without self-reported delayed vaccination; and b) with and without self-reported missing of vaccine dose.

a) Self-reported delayed vaccination										
**PACV-BR score**	**Group A – with**			**Group B – without**			**t**	**p-value**	**MD**	**MD 95%CI**
	n	Mean	SD	n	Mean	SD				
	30	34.17	24.99	80	10.48	14.96	4.87	<0.001	23.69	13.84–33.53
**b) Self-reported dose missing**										
**PACV-BR score**	**Group A - with**			**Group B - without**			**t**	**p-value**	**MD**	**MD 95%IC**
	n	Mean	SD	n	Average	SD				
	3	77.67	5.03	107	15.23	18.58	5.78	<0.001	62.43	41.05–83.81

SD: standard deviation; MD: mean difference.

**Figure 2:**
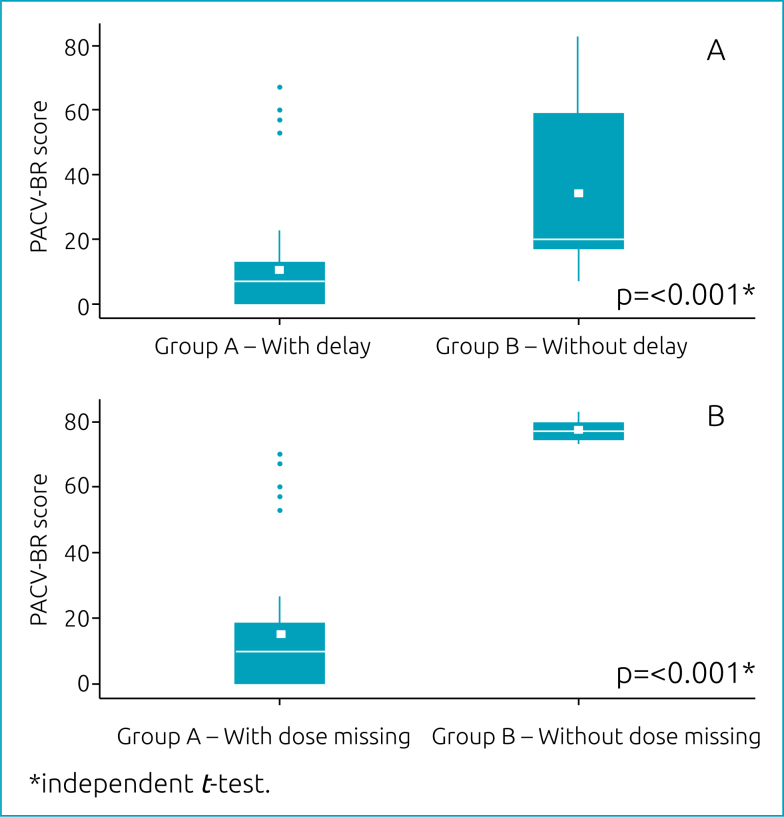
Mean *Parent Attitudes about Childhood Vaccine* — Brazilian version scores of parents a) with and without self-reported delayed vaccination; and b) with and without self-reported missing of vaccine dose.

The mean PACV-BR score difference between the groups of parents that reported delaying at least one of the regular scheduled doses of childhood vaccines was 23.6 times (CI95% 13.84–33.53). Accordingly, the mean score difference between parents that refused at least one vaccine dose and those that did not report this behavior was 62.43 times (CI95% 41.05–83.81) (Figure 2).

## DISCUSSION

Parental vaccine hesitancy evaluation scores, using the PACV's version transculturally adapted to Brazilian Portuguese, showed that 15.5% (n=17) of the research participants exhibited vaccine hesitancy, that is, had a score ≥50 in the PACV-BR. Likewise, in the validation study of the PACV's original version, made in Seattle, USA, this percentage was 15.1% (n=47) from a sample of 310 parents.^4^

In a study that evaluated vaccine trust in Brazil using a general questionnaire of the parents’ opinion about vaccines, among the 952 interviewees, the general rate of vaccine hesitancy was 16.5% (n=157).^12^ Accordingly, another national evaluation revealed that the percentage of interviewees that were not vaccinated or did not vaccinate a child under their care was 13% (n=260) out of 2,002 interviewees.^13^ In the pre-test of the Portuguese version of the PACV, 10% of the respondents had an average score ≥50 on the instrument score.^7^

Therefore, the percentage of hesitant Brazilian parents of this study, calculated by the PACV-BR's score, was close to the values of previous studies. These, however, were based on self-reported delaying and/or missing of doses and were developed, as the present study, in a sample of the Brazilian population, in a given territory. Consequently, they did not necessarily represent the country's entire population — and that is why we believe the variations presented here are entirely justifiable.

Another aspect to consider is the methodological design adopted in previous studies to obtain the parental vaccine hesitancy status. Such studies employed self-reported confirmations, answering a single dichotomous assertive question (yes; no) about the vaccine situation/behavior. As for the vaccine hesitancy status adopted in the present study, it was obtained in a structured manner and by answering to the questions (items) of the PACV-BR tool.

None of the PACV-BR^7^ items was removed in this study. The 15 items of the tool adapted to Brazilian Portuguese were validated in two factors, fulfilling the conditions to perform the EFA, Bartlett's significant test of sphericity, and the KMO with acceptable quality — in addition to the total variation above 60.0%, at least three items withheld in each factor, factorial loads above 0.4, and dimension eigenvalues >1.0. These parameters fulfilled the conditions indicated in the literature to validate the tool's construction^9,10^ and, consequently, to attest that the PACV-BR's structure is suitable and can summarize or explain the set of variables observed.^14^ The EFA identified that the PACV-BR's items “go together,” that is, the variables have the same underlying structure and can be translated into factors or latent dimensions that summarize or explain the set of items observed.^14,15^

The two latent dimensions that explain the PACV-BR's set of variables observed, respectively called “behaviors” and “beliefs,” have eight items related to the parents’ behavior regarding immunization and seven items related to parents’ beliefs and convictions about vaccines. This component's nominal structure was established by the analysis of the tool's item content, of its semantic relationships, and of the level of similarity expressed in each affirmative. In this step, a dialog with the “3Cs” model of vaccine hesitancy determinants was pursued, as proposed by the WHO. This model grouped into three main categories the reasons that cause vaccinate hesitancy in individuals, namely: complacency, confidence, and convenience.^1,2^

The construct and reliability validation study of the PACV's original version in English identified three domains using EFA — behavior; safety and effectiveness; and general attitudes — that explained 70% of the primary tool's total variation.^9^ Nevertheless, in our study, the items of the PACV translated and culturally adapted to Brazil were grouped by the factor study, in the present study, into two domains — a different structure from the one suggested by Opel et al.^4^ The maintenance of both domains identified for the PACV-BR in the present study was based on the cut-off value (>0.40) of the factorial loads presented by the items as a result of the EFA in the components’ rotated analysis^9,15^ and in the parameters mentioned above.

Accordingly, validation and transcultural adaptation studies of other tools developed in Brazil did not find the same factors seen in the original-language scale either.^16,17^ About this phenomenon, Pesce et al.^17^ say that the psychometric data of international constructs, tested in populations of different cultures, can actually be arranged differently for factor analysis. The authors claim the semantic, idiomatic, and cultural features of the population and/or the target language incorporated in the steps of transcultural adaptation to compensate the differences between both universes under study (origin language/culture; target language/culture) can change the dimension structure and, consequently, the relationships established between the items. This implies construct divergences that are eventually detected in the validation and evaluation of the psychometric properties of the tool undergoing cultural adaptation — which was the case here.

Reliability analysis of the PACV adapted to Brazil resulted in Cronbach's alpha values between 0.715 and 0.854 for the items, 0.918 for the tool as a whole, 0.877 for factor 1, and 0.825 for factor 2, in addition to an ICC of 0.984. These results are within the values deemed acceptable by the literature^9,10,15^ and are greater than those registered in the original reliability study of the English PACV, whose Cronbach's alpha values for the three primary factors (behavior; safety and effectiveness; and general attitudes) were 0.74, 0.84, and 0.74, respectively. It was not possible to compare the values of Cronbach's alphas by item and of the construct as a whole with the values found in the primary validation study in English, nor the ICC value, because the only information that the original publication has for the tool's three domains is Cronbach's alpha.^4,5^

In the comparison between groups, made to test the hypothesis that there is a difference of PACV-BR score means between the group of parents that reported delaying and/or missing vaccine doses and the parents that did not report delaying and/or missing doses, the PACV-BR means were considerably greater among the former. On the other hand, in the groups that did not delay and/or miss vaccine doses, the PACV-BR score mean was lower, which strengthens the validity of the PACV-BR's structure and gives evidence of validity of the PACV-BR's scores to differentiate hesitant and non-hesitant parents.

The latter result was expected by the research team, since there is a strong relationship between the total score of parents in the PACV and the immunization status of their children. In the PACV's primary validation research, in English, the parents that got higher total scores in the tool were significantly associated with a greater percentage of undervaccination of their children^6^.

This study has limitations. More specifically, it was limited to evaluate some psychometric parameters of validity and reliability. Therefore, we encourage the future study of other statistical parameters (for example, converging validity, discriminating validity, and confirmatory factor analysis) involving the PACV-BR.

## Data Availability

The database that originated the article is available with the corresponding author.

## References

[1] World Health Organization [homepage on the Internet] Strategic Advisory Group of Experts in Immunization (SAGE).

[2] Santos CJ, Carvalho-Neto AP, Rocha TJ, Costa PJ (2022). Vaccine hesitation and the ‘pandemic’ of the unvaccined: what to do to face the new “Vaccine Revolt”?. Medicina (Ribeirão).

[3] World Health Organization [homepage on the Internet] Ten threats to global health in 2019.

[4] Opel DJ, Taylor JA, Zhou C, Catz S, Myaing M, Mangione-Smith R (2013). The relationship between parent attitudes about childhood vaccines survey scores and future child immunization status: a validation study. JAMA Pediatr.

[5] Opel DJ, Taylor JA, Mangione-Smith R, Solomon C, Zhao C, Catz S (2011). Validity and reliability of a survey to identify vaccine-hesitant parents. Vaccine.

[6] Opel DJ, Mangione-Smith R, Taylor JA, Korfiatis C, Wiese C, Catz S (2011). Development of a survey to identify vaccine-hesitant parents: the parent attitudes about childhood vaccines survey. Hum Vaccine.

[7] Santos CJ, Costa PJ (2022). Adaptação transcultural e validação para o Português (Brasil) do Parent Attitudes About Childhood Vaccine (PACV). Ciên Saúde Coletiva.

[8] Costa ZM, Pinto RM, Mendonça TM, Silva CH (2020). Brazilian validation of the item banks on Sleep Disturbance and Wake Disturbance in the Patient-Reported Outcomes Measurement Information System (PROMIS). Cad Saúde Pública.

[9] Figueiredo DB, Silva JA (2010). Visão além do alcance: uma introdução à análise fatorial. Opin Publica.

[10] Pasquali L (2013). Psicometria: teoria dos testes na psicologia e na educação.

[11] Fleiss JL, Levin B, Paik MC (2003). Statistical methods for rates and proportions.

[12] Brown AL, Sperandio M, Turssi CP, Leite RM, Berton VF, Succi RM (2018). Vaccine confidence and hesitancy in Brazil. Cad Saúde Pública.

[13] Avaaz.org [homepage on the Internet] As Fake News estão nos deixando doentes?.

[14] Hair JF, Black WC, Babin BJ, Anderson RE, Tatham RL, Gouvêa MA (2009). Análise multivariada de dados.

[15] Tabachnick BG, Fidell LS (2018). Using multivariate statistics.

[16] Aroian KJ, Schappler-Morris N, Neary S, Spitzer A, Tran TV (1997). Psychometric evaluation of the Russian language version of the resilience scale. J Nurs Meas.

[17] Pesce RP, Assis SG, Avanci JQ, Santos NC, Malaquias JV, Carvalhaes R (2005). Cross-cultural adaptation, reliability and validity of the resilience scale. Cad Saúde Pública.

